# Genome-wide analysis of long noncoding RNA expression profile in nasal mucosa with allergic rhinitis

**DOI:** 10.1186/s12920-021-00949-4

**Published:** 2021-04-09

**Authors:** Xian Wei, Man Xu, Chao Wang, Shengjian Fang, Yu Zhang, Weihua Wang

**Affiliations:** 1 Department of Otolaryngology-Head and Neck Surgery, Shanghai East Hospital, Tongji University School of Medicine, 150 Jimo Road, Shanghai, 200120 People’s Republic of China; 2 Department of Otolaryngology-Head and Neck Surgery, Shanghai East Hospital Ji’an Hospital, Ji’an, Jiangxi People’s Republic of China

**Keywords:** Allergic rhinitis, Microarray, Expression profile, Long non-coding RNA, CXCL12, CXCR4

## Abstract

**Background:**

Long noncoding RNAs (lncRNAs) are involved in a variety of human immune diseases. However, the expression profile and precise function of lncRNAs in allergic rhinitis (AR) remain unknown. In the present study, genome-wide analysis of lncRNA expression was performed in the nasal mucosa tissue and mRNA regulatory relationship was examined among patients with or without AR.

**Methods:**

Microarray assays were performed and the differential expressions of lncRNAs or mRNA were verified through RT-PCR. The lncRNA functions were annotated using Gene Ontology (GO) and Kyoto Encyclopedia of Genes and Genomes (KEGG). The potential regulatory relationships between lncRNAs and the co-expressed mRNAs were analyzed using Cytoscape software. The expressions of specific lncRNAs and mRNAs were examined using an in vitro cell model.

**Results:**

A total of 57 lncRNAs and 127 mRNAs were dysregulated in the nasal mucosa tissue of patients with AR, compared to those of patients without AR (fold change > 2.0 and *P* < 0.05). GO and pathway analysis indicated that the lncRNA–co-expressed mRNAs were enriched in several biological processes and cellular signaling pathways related to AR, such as positive regulation of the integrin biosynthetic process, cell adhesion, and leukocyte transendothelial migration. Some lncRNAs regulated the co-expressed genes in a cis- and/or trans-regulatory manner. Furthermore, allergen exposure significantly increased the expression of lnc-CXCL12-4, CXCL12, and CXCR4 in BEAS-2B cells compared to untreated cells (*P* < 0.01).

**Conclusion:**

The results of the present study suggest that lncRNAs participate in the biological pathways related to AR. Leukocyte transepithelial migration may be a potential target for lncRNAs to regulate allergic inflammation and CXCL12/CXCR4 axis plays an important role in the inflammatory process of AR.

**Supplementary Information:**

The online version contains supplementary material available at 10.1186/s12920-021-00949-4.

## Background

Allergic rhinitis (AR) is an IgE-mediated upper airway inflammatory response, which is becoming a global health problem [1]. AR is characterized by symptoms of congestion, rhinorrhea, sneezing, and itching. Because of the identity of the upper and lower airway allergic inflammatory responses, 40% of AR patients have or will have asthma [2]. AR seriously affects the quality of life of patients and increases the family and social economic burden [1, 3]. Like other allergic diseases, the etiology of AR has complex components. A growing body of evidence indicates that the imbalance of the Th1/Th2 immune response contributes to the onset of AR [4–6]. However, the underlying pathogenesis of AR remains unclear.

It is well known that most of the genome is transcribed into RNA, but only a very small percentage of the transcripts are protein-coding genes, accounting for only 1.5–2% [7]. Therefore, there has been a growing interest in the role of noncoding RNAs. Long noncoding RNAs (lncRNAs) are a group of RNA molecules with transcription lengths of more than 200 nucleotides which do not encode any protein products [8]. LncRNAs widely participate in regulatory functions at the epigenetic, transcriptional, and post-transcriptional levels [9]. Although aberrantly expressed lncRNAs have been detected in nasal mucosa with AR in human and animal models [10–12], studies on the roles of lncRNAs are still at a preliminary stage. The expression pattern and function prediction of lncRNAs in AR remain unclear.

The aim of the present study is to examine the expression profiles of lncRNAs and mRNAs in AR. We identified the differential expression of lncRNAs and mRNAs in nasal tissues from AR and non-AR patients using microarray assays. Moreover, we analyzed the potential functions of the differentially expressed lncRNAs via bioinformatics analysis and validated the meaningfully enriched pathway using an in vitro cell culture model.

## Materials and methods

### Patients and tissue collection

A total of 8 AR patients and 10 non-AR patients were admitted to the Department of Otolaryngology-Head and Neck Surgery, Shanghai East Hospital between 2016 and 2019. All of the patients with AR had a positive skin prick test reaction only to dust mites and were diagnosed based on their medical history, nasal endoscopic examination, and allergen skin prick test. None of the participants had received topical or systemic glucocorticoid therapy for 4 weeks before tissue collection. Nasal mucosal tissues were obtained surgically from the inferior turbinates of the patients. The harvested samples were snap-frozen in liquid nitrogen and stored at –80℃. All patients had nasal septum deviation and were scheduled to undergo septoplasty and partial removal of the inferior turbinates. The study conforms to the standards of the Declaration of Helsinki. Patients who had a history of previous nasal surgery, smoking, autoimmune diseases, concurrent sinusitis, or systemic diseases were excluded from our study. Patient clinical characteristics are summarized in Additional file 1.

### Total RNA extraction

For the lncRNA and mRNA microarrays, total RNA was extracted from 100 mg of nasal mucosal tissue from 3 AR patients and 3 non-AR patients using TRIzol reagent (Invitrogen, Carlsbad, CA, USA). Total RNA was quantified by a NanoDrop ND-2000 spectrophotometer (Thermo Fisher Scientific, Wilmington, DE, USA) and the RNA integrity was assessed using an Agilent 2100 Bioanalyzer (Agilent Technologies, Santa Clara, CA, USA).

### LncRNA chip microarray

Total RNA labeling, microarray hybridization, and washing were performed using an Affymetrix Human OE lncRNA array (Affymetrix, Santa Clara, CA, USA) based on the manufacturer’s instructions. The microarray profiling was conducted in the laboratory of Shanghai OEBiotech (Shanghai, People's Republic of China). This microarray contains probes for 25,986 mRNAs and 66,741 lncRNAs.

### Data analysis

Raw data were extracted using the Affymetrix GeneChip Command Console (version 4.0, Affymetrix). RMA (Robust Multichip Average) normalization for both gene and exon level analysis was performed using Expression Console (version1.3.1, Affymetrix). GeneSpring software (version 13.1, Agilent Technologies) was employed to complete subsequent data processing. After log2 transformation of the raw signals, differential expression of lncRNAs and mRNAs was defined by the absolute value of fold change (> 2.0) and *P* value < 0.05 (Student’s *t*-test). The unsupervised hierarchical clustering of differentially expressed lncRNAs and mRNAs was carried out. The differentially expressed mRNAs were input into the DAVID database (http://david.abcc.ncifcrf.gov) for Gene Ontology (GO) and Kyoto Encyclopedia of Genes and Genomes (KEGG) pathway annotation classification.

### Quantitative RT–PCR validation

Total RNA was extracted from the nasal mucosal tissue from 8 AR and 10 non-AR patients using TRIzol reagent (Invitrogen) according to the manufacturer's instructions. The first strand cDNA was reverse-transcribed from 500 ng of total RNA using PrimeScript™ RT Master Mix (Takara Bio, Inc., Otsu, Japan). SYBR Premix Ex Taq™ (Takara Bio, Inc.) was used to conduct real-time PCR using an ABI 7500 Real-Time PCR System (Applied Biosystems, Foster City, CA, USA). The specific primer sequences used in qRT–PCR are shown in Table 1. The expression levels of lncRNAs and mRNAs were normalized to glyceraldehyde 3-phosphate dehydrogenase (GAPDH) and quantified using the 2^−ΔΔct^ method.

**Table 1:** Primers used for qRT–PCR of lncRNA and mRNA expression

lncRNA/mRNA	Forward primer (5′–3′)	Reverse primer (5′–3′)
lnc-CBR1-3	GGTCAAGCCAAGCCAACAG	GTCCTCAGCACCACTTATTAGT
lnc-HPR-1	GAGGCACAGACAGGTTGAGTA	CGGTCACAGCCAAGCAGTA
lnc-SEPT7L-5	CCATGCCATTCAGGTCCAATC	GGAATGGATGCGAATGGAATGA
DLGAP1-AS1	TATCTGAGAGCCAGCGAACTT	CTTCATAGCCTGTTGCGTCAT
lnc-CXCL12-4:1	GACCGCTCCCGCCTAATG	TGCTTAGCCCTCCGGATACC
lnc-CXCL12-4:2	CTACAGATGCCCATGCCGAT	AGGTTGGACACTTGGCTTGT
MUC7	GCTTGCTTCTCGTTCAGT	GTGATGATGCCTTCTGTGA
IGFBP3	GTCCTCCTTAGCACAATGTA	TCCTCCTTCCTGTTCTGATA
CXCL12	GACAAGTGTGCATTGACCCG	GCCCTTCCCTAACACTGGTT
CXCR4	AACTTCAGTTTGTTGGCTGCG	GATCCCCTCCATGGTAACCG
GAPDH	TGTTGCCATCAATGACCCCTT	CTCCACGACGTACTCAGCG

### Cell culture and allergen treatment

Human bronchial epithelial cell line BEAS-2B was purchased from ATCC (Walkersville, MD, USA) and cultured in BEGE medium with a Bullet Kit (Lonza, Walkersville, MD, USA). All incubations were carried out in a humidified atmosphere of 5% CO_2_ at 37℃. All experiments were performed with BEAS-2B cells at 80–90% confluency. Cells were exposed to different concentrations of ovalbumin (OVA) (100 μg/mL) or house dust mite (HDM) (Wolwo Bio-Pharmaceutical, Zhejiang, China) (40 μg/mL) for 4 h.

### LncRNA–mRNA co-expression analysis

Before predicting the possible functions of lncRNAs in AR, a correlation analysis of lncRNAs and mRNAs involved in allergic inflammation was carried out [13]. According to the normalized signal intensity of each differentially expressed lncRNA and mRNA in this microarray assay, Pearson’s Correlation Coefficient (PCC) of their expression was calculated to evaluate the correlation between lncRNAs and mRNAs. The co-expressed mRNAs of lncRNAs were identified by *P* values of PCC < 0.05 and absolute values of PCC > 0.8.

### Functional enrichment analysis of the lncRNAs

For function prediction of lncRNAs, an enrichment analysis of the co-expressed mRNAs was performed using the hypergeometric cumulative distribution function [13]. The enriched annotations of GO and KEGG pathways were assigned to the corresponding lncRNA as its predicted functions. The threshold of statistical significance was set as *P* < 0.05 and false discovery rate (FDR) < 0.01. The most enriched annotations reflected the potential functions of the co-expressed lncRNAs.

### LncRNA–mRNA regulatory network analysis

To explore the potential target genes in AR, cis- and trans-regulatory analysis of the differentially expressed lncRNAs was performed. For cis-regulatory analysis, we identified the cis-regulated genes when the co-expressed mRNA loci were within 100 kbp upstream and downstream of the given lncRNA. Another regulatory mechanism of the specific lncRNAs in the expression of certain genes involves the factors mediating chromatin transcription (TFs) [13, 14]. So for transcriptional factor correlation analysis, the hypergeometric cumulative distribution function was used to compare the co-expressed mRNAs with the genes regulated by certain TFs (*P* < 0.05 and FDR < 0.01). Then we predicted that these lncRNAs possibly regulated the target genes in a trans-regulatory manner. The lncRNA–TF–mRNA network was constructed based on the interactions between the lncRNAs and the co-expressed target mRNAs. We selected the top 10 prediction regulating relationships with the highest prediction reliability to construct the core network map using Cytoscape software.

### Statistical analysis

All data were expressed as mean ± standard deviation and analyzed using IBM SPSS Statistics, Version 22 (IBM Corp., Armonk, NY, USA). The differences in expression of lncRNAs and mRNAs in nasal mucosal tissue between AR and non-allergic patients were analyzed using Student's *t*-tests. *P* < 0.05 was considered statistically significant.

## Results

### Differentially expressed lncRNAs and mRNAs in AR

**Fig. 1:**
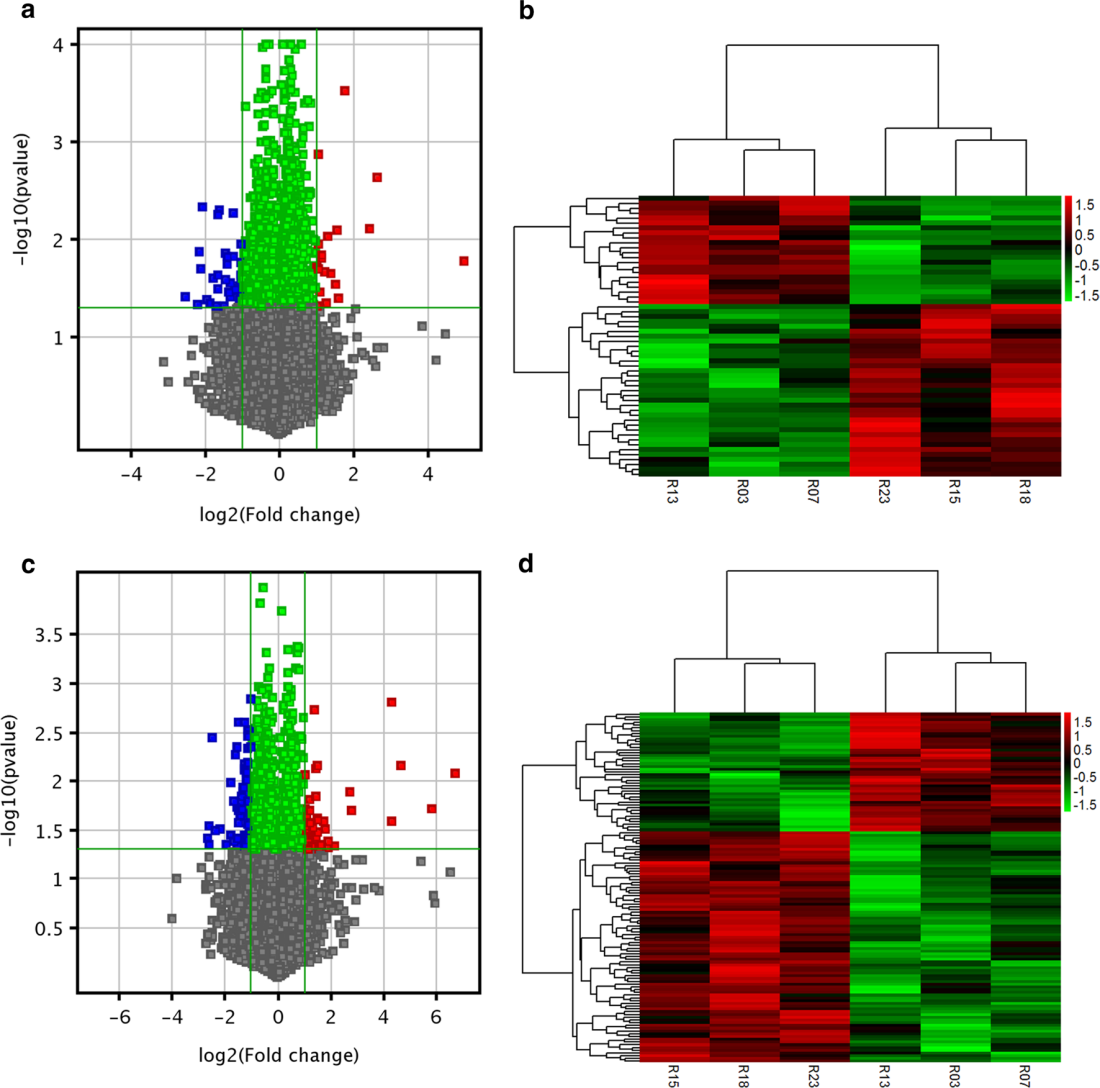
Differential expression of lncRNAs and mRNAs in the nasal mucosa from AR and non-AR patients. Volcano plots show the differentially expressed lncRNAs (**a**) and mRNAs (**c**). The vertical lines correspond to 2.0-fold up and down, and the horizontal lines represent *P* values = 0.05. Heat maps indicate hierarchical clustering results of differentially expressed lncRNAs (**b**) and mRNAs (**d**). Each row corresponds to one lncRNA or mRNA, and each column represents one sample. Red and green colors indicate upregulated and downregulated expression, respectively. AR samples: R03, R07 and R13; non-AR samples: R15, R18, and R23

We profiled the expression patterns of lncRNAs and mRNAs associated with AR via microarrays. Our data showed that a total of 57 lncRNAs were differentially expressed in the nasal mucosa from AR and non-AR patients, with 22 upregulated and 35 downregulated, as indicated by the volcano plots and heat maps (Fig. 1a, b). Simultaneously, we found that a total of 127 mRNAs were differentially expressed, with 43 mRNAs upregulated and 84 mRNAs downregulated in the nasal mucosa from patients with AR compared to those without AR (Fig. 1c, d) (see Additional file 2 for the differentially expressed mRNAs and lncRNAs). The top 20 differentially expressed lncRNAs and mRNAs are included in Table 2. According to the absolute value of fold change (FC), lnc-MUC7-1 and lnc-AC011294.3.1–6 are the most upregulated and downregulated lncRNAs, respectively. MUC7 and IGFBP3 are the most upregulated and downregulated mRNAs, respectively. These results indicate that these lncRNAs and mRNAs may have specific functions in the development of AR.

**Table 2:** Top 20 upregulated and downregulated lncRNAs and mRNAs in the nasal mucosa tissue from patients with AR compared to without AR

Upregulated lncRNAs		Downregulated lncRNAs		Upregulated mRNAs		Downregulated mRNAs	
lncRNA	FC (abs)	lncRNA	FC (abs)	mRNA	FC (abs)	mRNA	FC (abs)
lnc-MUC7-1	31.7086	lnc-AC011294.3.1-6	5.8161	MUC7	100.7255	IGFBP3	6.3291
lnc-UMOD-2	6.2684	lnc-SERPINB12-3	4.5107	HP	56.9782	ROBO2	6.0832
lnc-HPR-1	5.4470	lnc-CBR1-3	4.3943	GP2	25.1581	THY1	5.9538
lnc-FADD-2	3.3699	lnc-F5-1	4.3442	HPR	19.7971	SELP	5.5017
lnc-AC073416.2-2	3.0700	lnc-TACC2-7	4.1595	IGHD3-10	19.2196	C1QTNF3	5.1029
lnc-HTN1-1	2.9277	lnc-XRCC4-6	3.8426	SMR3B	6.8644	FMO2	4.6298
lnc-STOM-7	2.9026	lnc-TAS2R1-24	3.6408	PIP	6.4510	MGARP	3.8910
lnc-DNAJC3-1	2.5966	lnc-FRG2C-15	3.3977	CA2	4.3661	FAM72C	3.4443
lnc-ADAM7-1	2.4617	lnc-ADAMTS1-2	3.1879	EFCAB4B	3.7810	IER3	3.3861
lnc-GPAT2-6	2.3813	lnc-TMEM207-2	3.0959	CRISP2	3.6558	PLXDC1	3.2197
lnc-CALM2-10	2.3379	lnc-IGIP-2	3.0957	BHLHA15	3.4208	GPR18	3.1555
lnc-CCDC90B-3	2.2304	lnc-FAM185A-3	3.0948	ALDH1L1	3.3048	CCDC169	3.0557
lnc-SEPT7L-5	2.2265	lnc-AC023469.1.1-2	3.0728	PAIP2B	3.0549	CNTNAP3B	2.9703
lnc-FDFT1-1	2.1435	lnc-GUCY1A2-1	2.7717	TMEM56	2.8856	GLI3	2.9433
lnc-TPPP2-1	2.1042	lnc-SERPINB12-1	2.7467	FCGR3B	2.8654	CXCL12	2.9255
lnc-GCGR-1	2.0608	lnc-FMO6P-2	2.7096	PYGB	2.8527	CLDN1	2.8886
lnc-DLG5-2	2.0601	lnc-C10orf68-7	2.6243	ANO5	2.8427	RMI2	2.8873
lnc-SLC6A18-1	2.0334	lnc-MARCKS-7	2.6001	C10orf90	2.8226	STON2	2.8486
lnc-ATXN7-9	2.0315	lnc-GMPS-5	2.5285	GGTA1P	2.7631	EYA1	2.8337
lnc-ME1-2	2.0308	DLGAP1-AS1	2.5205	ANO1	2.7066	CAPN5	2.8296

FC (abs): absolute fold change

### Validation of the microarray data by qRT–PCR

**Fig. 2:**
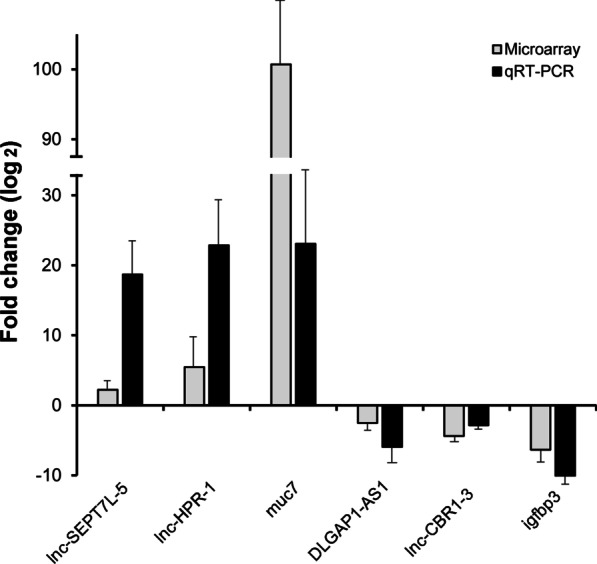
Quantitative RT–PCR validation of the microarray analysis. The expression levels of the randomly selected 4 lncRNAs and 2 mRNAs were validated by real time RT–PCR. The heights of the columns represent the mean values of fold changes (log_2_) in expression for the lncRNAs and mRNAs. Fold change is positive when the expression is upregulated and negative when downregulated. The expression levels of lncRNAs and mRNAs were normalized to glyceraldehyde 3-phosphate dehydrogenase (GAPDH)

To validate the microarray results, we randomly chose 4 lncRNAs and 2 mRNAs from the differentially expressed lncRNAs and mRNAs for qRT–PCR. As Fig. 2 shows, our data indicated that the expression trend of the selected RNAs was consistent with the results of the microarray analysis.

### GO and KEGG analysis of the differentially expressed lncRNAs

**Fig. 3:**
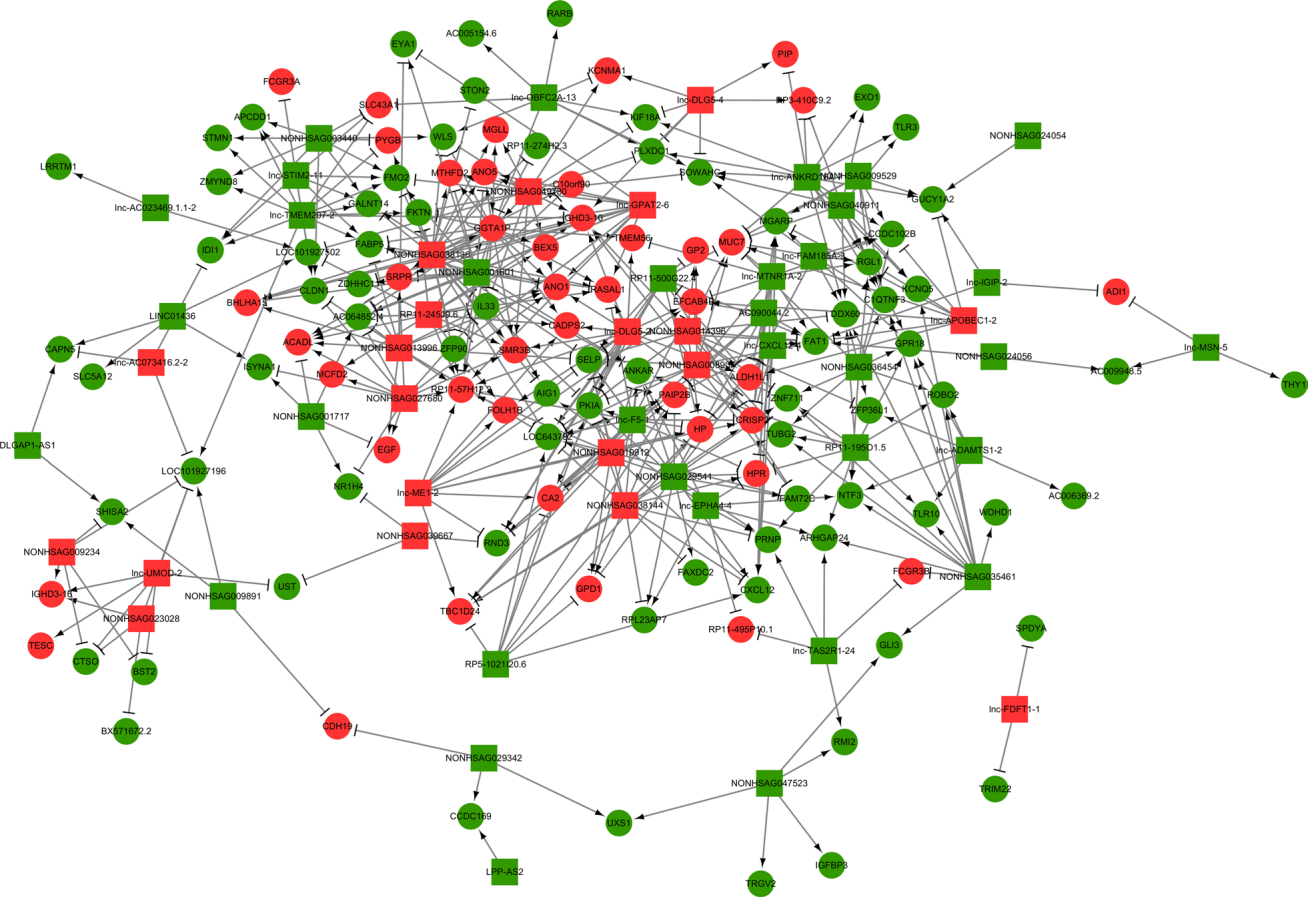
LncRNA–mRNA co-expression network. The square nodes represent lncRNAs, and the round nodes represent mRNAs. The red and green colors indicate high and low expression, respectively. The lines with arrowheads or blunt ends represent positive or negative regulation, respectively

There are thousands of co-expression relationships between the differentially expressed lncRNAs and mRNAs. We analyzed the co-expression relationships of the top 500 pairs by PCC and constructed a co-expression network using Cytoscape software (Fig. 3). The visible network also indicated that one lncRNA could regulate the expression of multiple mRNAs, and the expression of the same gene could be regulated by multiple lncRNAs.

**Fig. 4:**
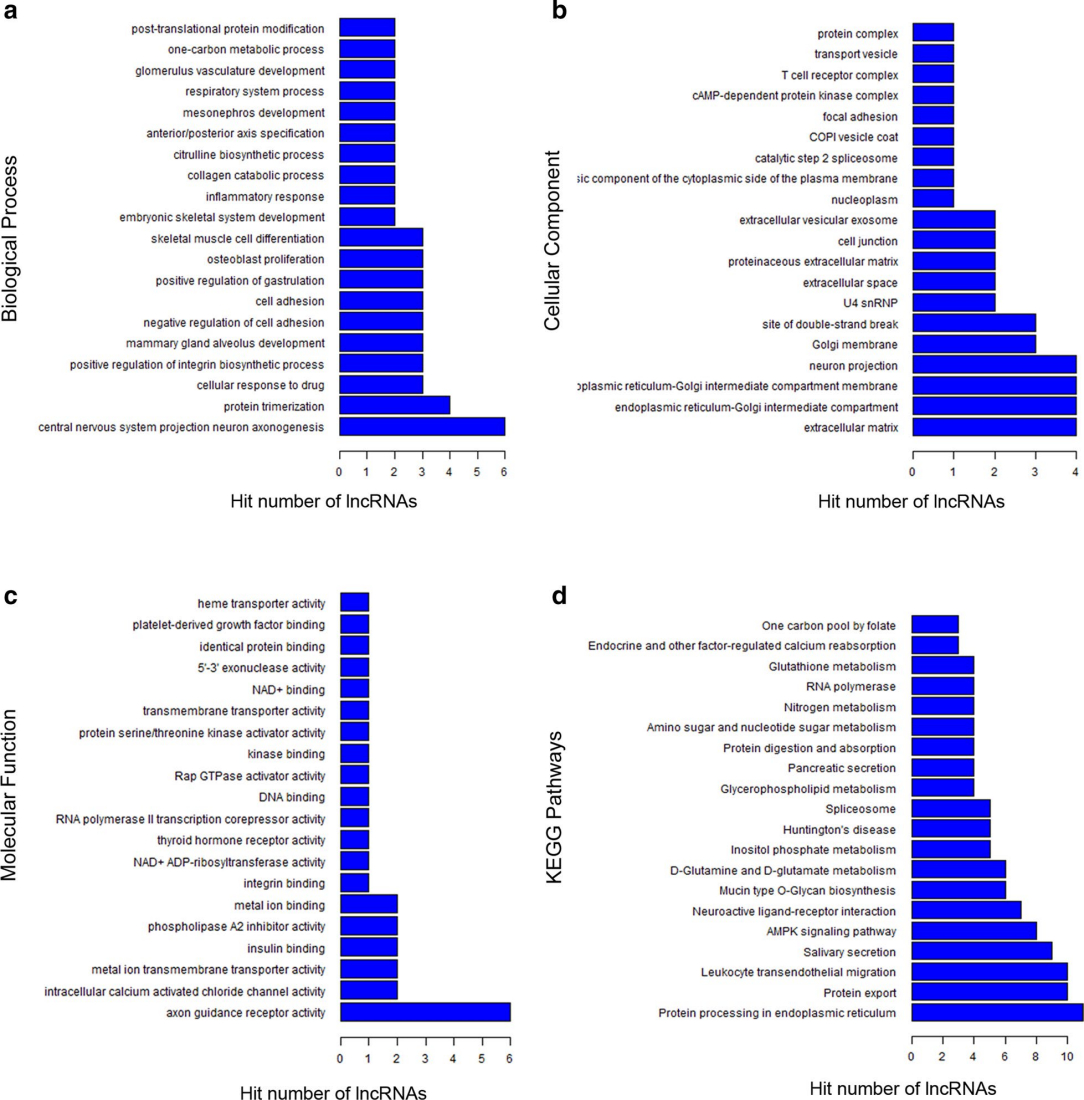
Top 200 hits of GO annotations and KEGG pathways of the co-expressed mRNAs with the differentially expressed lncRNAs. GO functional enrichment analysis (**a**–**c**) and KEGG pathway enrichment analysis (**d**) are performed, respectively. The ontology covers three domains: biological process (**a**), cellular component (**b**), and molecular function (**c**). The x-axis shows the hit number of lncRNAs annotated, and the y-axis shows the GO annotations or pathways

The potential functions of the lncRNAs were predicted by the GO and KEGG pathway annotations of their co-expressed mRNAs. The GO categories are biological process, molecular function, and cellular component. GO analysis of the co-expressed mRNAs revealed that the most enriched annotations were involved in positive regulation of the integrin biosynthetic process, cell adhesion, focal adhesion, inflammatory response, extracellular matrix, T cell receptor complex, cell junction, and intracellular calcium activated chloride channel activity. We counted and summarized the top 20 GO annotations with the most credentiality (Fig. 4a–c). In addition, the KEGG pathway analysis indicated that protein processing in the endoplasmic reticulum, protein export, the MAPK signaling pathway, and leukocyte transendothelial migration were the most frequently predicted pathways (Fig. 4d). These pathways are associated with immune cell proliferation and migration. The results indicate that these differentially expressed lncRNAs may play important roles in the pathophysiological process of allergic inflammation, such as inflammation, cell differentiation, proliferation, and chemotactic movement.

### Analysis of the lncRNA–mRNA regulatory network

LncRNAs may regulate the nearby genes in a cis-regulatory manner. Therefore, we screened the chromosomal co-expressed mRNAs 100 kbp upstream and downstream of 57 differentially expressed lncRNAs and identified 35 lncRNAs with 41 potential cis-regulated mRNAs. The lncRNAs and the potential cis-regulated mRNAs are included in Table 3.

**Table 3:** LncRNAs and the potential cis-regulated mRNAs

Chrom	Correlation	lncRNA	mRNA
5	0.987369	lnc-XRCC4-6	VCAN
5	0.986823	lnc-IGIP-2	CTB-131B5.2
3	0.986734	lnc-TMEM207-2	CLDN1
16	0.986412	lnc-HPR-1	HP
2	0.983264	lnc-CALM2-10	MCFD2
1	0.981938	lnc-FMO6P-2	FMO2
1	0.981073	lnc-F5-1	SELP
10	0.97774	lnc-DLG5-4	KCNMA1
7	0.974997	lnc-FAM185A-3	LRRC17
10	0.966048	lnc-CXCL12-4	CXCL12
4	0.964856	lnc-MTNR1A-2	FAT1
9	0.964309	lnc-STOM-7	GGTA1P
4	0.96054	lnc-STIM2-11	PCDH7
10	0.950211	lnc-C10orf68-7	C10orf68
11	0.94865	lnc-GUCY1A2-1	GUCY1A2
2	0.946103	lnc-LYPD1-2	NCKAP5
16	0.945882	lnc-UMOD-2	GP2
16	0.944974	lnc-HPR-1	HPR
5	0.941399	lnc-TAS2R1-24	SEMA5A
11	0.932664	lnc-CRTAM-1	C11orf63
6	0.931226	lnc-ME1-2	PGM3
2	0.92093	lnc-AC023469.1.1-2	RND3
4	0.917134	lnc-MUC7-1	MUC7
–	0.916819	lnc-MARCKS-7	BMS1P6
21	0.910659	lnc-ADAMTS1-2	ADAMTS5
11	0.904913	lnc-FADD-2	ANO1
4	0.891984	lnc-MUC7-1	SMR3B
1	0.890773	lnc-C8B-1	DAB1
9	0.886791	lnc-ANKRD18A-7	CNTNAP3
2	0.88018	lnc-OBFC2A-13	MYO1B
–	0.863629	lnc-MARCKS-7	RP11-782C8.5
4	0.858795	lnc-MUC7-1	SMR3A
2	0.856086	lnc-EPHA4-4	EPHA4
1	0.837189	lnc-RAVER2-1	CACHD1
4	0.834533	lnc-MUC7-1	AMBN
13	0.829561	lnc-DNAJC3-1	DNAJC3
3	0.82015	lnc-CNTN6-2	AC090044.2
21	–0.8336	lnc-CBR1-3	RIMKLBP1
–	–0.85488	lnc-SEPT7L-5	BMS1P6
12	–0.87456	lnc-APOBEC1-2	APOBEC1
–	–0.88594	lnc-SEPT7L-5	RP11-782C8.5
9	–0.9127	lnc-STOM-7	RP11-477J21.6

**Fig. 5:**
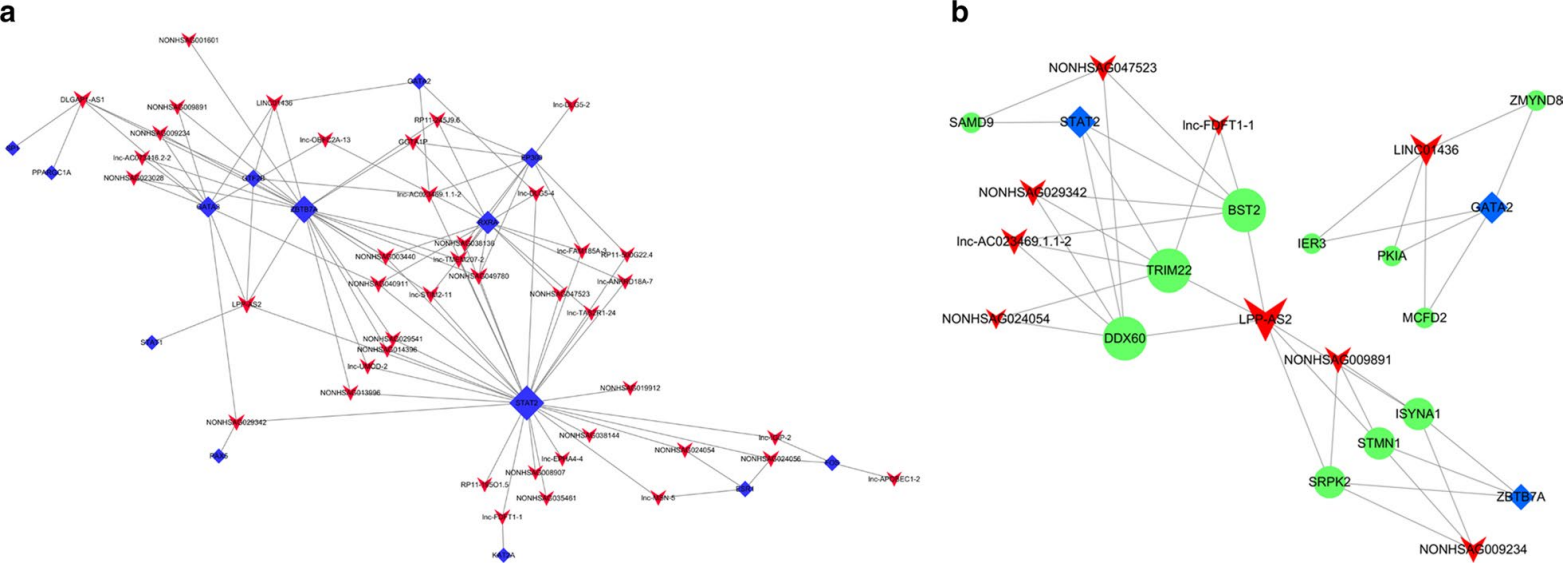
The core network of trans-regulatory analysis with the differentially expressed lncRNAs. The top 100 lncRNA-transcription factor (TF) pairs with the most credentiality were selected. The lncRNA–TF two-element networks (**a**) and the lncRNA–TF–mRNA three-element networks (**b**) are constructed. The red arrowhead nodes represent lncRNAs, the blue rhombus nodes represent TFs, and the green round nodes represent mRNAs

We calculated the significance of enrichment of each co-expressed mRNA in TFs from the Encyclopedia of DNA Elements, and identified 143 lncRNA–TF pairs, including 50 lncRNAs and 47 TFs. We selected the top 100 lncRNA–TF pairs with the most credentiality and created the lncRNA–TF two-element network relationship using Cytoscape software (Fig. 5a). Adding the above-mentioned co-expressed mRNA, we created the lncRNA–TF–mRNA three-element network relationship. The visible core network map was generated based on the top 10 lncRNA–TF–mRNA pairs (Fig. 5b). As shown in the network map, LPP-AS2 is the regulatory lncRNA with most potential in the trans-regulation of the target mRNAs.

### Expression of lnc-CXCL12-4 in airway epithelial cells after allergen stimulation

**Fig. 6:**
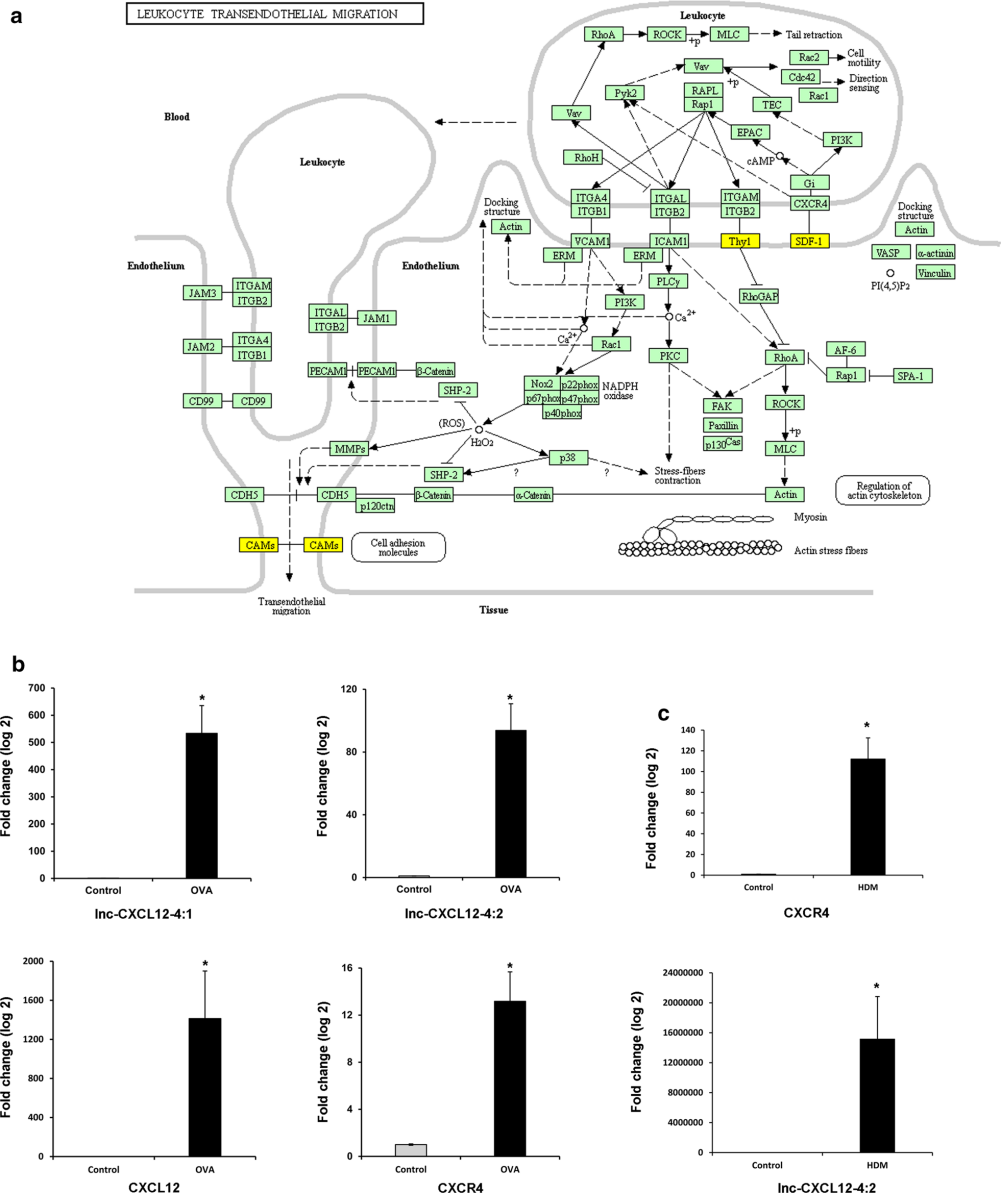
Differentially expressed lncRNAs in airway epithelial cells after allergen stimulation. **a** Diagram of the Leukocyte Transendothelial Migration pathway [33]. Three dysregulated mRNAs were associated with the Leukocyte Transendothelial Migration pathway in our microarray analysis. They are CXCL12 (also known as stromal cell-derived factor-1α, SDF-1α), THY1, and CLDN1 (shown as CAMs). Their positions in the Leukocyte Transendothelial Migration pathway have been highlighted in yellow. **b** Quantitative expressions of lnc-CXCL12-4, CXCL12, and CXCR4 were assessed by real-time RT–PCR from BEAS-2B cells treated with or without 100 μg/mL of OVA for 4 h. **c** Quantitative expressions of lnc-CXCL12-4 and CXCR4 were assessed by real-time RT–PCR from BEAS-2B cells treated with or without 100 μg/mL of OVA for 4 h. All qRT-PCRs were performed in triplicate, and the Δct values were calculated by using glyceraldehyde 3-phosphate dehydrogenase as the endogenous control. OVA: ovalbumin; *P < 0.01

Based on the KEGG pathway analysis, we know that leukocyte transendothelial migration is one of the frequently enriched pathways in our functional predictive analysis (Fig. 4d). From the DAVID database, we identified three differentially expressed mRNAs enriched in this pathway, and they are CXCL12 (also known as stromal cell-derived factor-1 α, SDF-1α), THY1, and CLDN1 (Fig. 6a). The cis-regulatory analysis indicated that lnc-CXCL12-4 and CXCL12 are both from chromosome 10 (Table 3) and lnc-CXCL12-4 may regulate the expression of CXCL12. We established an in vitro experimental environment to mimic the interaction between allergen and airway epithelial barrier. The effects of allergen on the expression of lnc-CXCL12-4 and the related mRNA were evaluated using real-time RT–PCR in BEAS-2B cells. Lower expression levels of lnc-CXCL12-4, CXCL12, and CXCR4 were detected in the unstimulated cells. Four hours after OVA/HDM exposure, the expression levels of lnc-CXCL12-4, CXCL12, and CXCR4 were significantly increased compared to the untreated cells (*P* < 0.05) (Fig. 6b, c). These results indicate that allergen might induce the expression of lnc-CXCL12-4 at an early stage when allergens enter the airway epithelial barrier, and regulate the signal of the CXCL12/CXCR4 axis in epithelial cells.

## Discussion

LncRNAs are considered to be important regulators of cellular process, such as development, differentiation, and metabolism, through affecting gene expression and cell homeostasis [15, 16]. Evidence is accumulating that shows lncRNAs are involved in biological functions by interacting with other molecules, such as DNA [17], RNA [18], proteins [19], and metal ions [20]. Abnormal expression of lncRNAs is involved in the pathophysiological process of many diseases, including cancer, respiratory disease, and diabetes [21–23]. Recent studies have explored the expression levels of lncRNAs in upper airway allergic diseases [5, 10, 11]. Ma et al. showed that the expression profile of lncRNAs was altered in the CD4^+^ T cells from AR mice [11]. A change in expression of lncRNAs has been detected in nasal mucosa from patients with AR, but no more bioinformatics analysis was provided [10]. These studies indicate that lncRNAs are involved in the pathogenesis of AR. However, the function and mechanism of action of lncRNAs in AR remain unclear.

In the present study, we assessed genome-wide lncRNA expression patterns in the nasal mucosa from patients with and without AR by microarray analysis, and predicted their possible functions by analyzing the co-expressed mRNAs. Moreover, we also used an in vitro model mimicking the allergen exposure environment of airway epithelium to verify the predicted results. Our results indicated that 57 lncRNA and 127 mRNA transcripts were identified as being differentially expressed between the two groups, including 22 upregulated and 35 downregulated lncRNAs, and 43 upregulated and 84 downregulated mRNAs, respectively. The correlation between some of the differentially expressed mRNAs and AR has been reported, such as ANO1, THY1, CXCL12, and IL33. The expression of ANO1 is higher in AR patients than in healthy controls and hypersecretion of fluid and mucus in AR is closely related to ANO1 [24]. THY1 gene expression was significantly increased in nasal mucosa tissues of AR mice [25]. Expression of CXCL12 in nasal mucosa of seasonal allergic rhinitis patients with asthma was up-regulated predominantly, compared with that in seasonal allergic rhinitis patients without asthma [26]. Serum level of IL-33 in patients with AR was significantly higher than in controls and can be used as a marker of the severity of AR [27]. These genes may be involved in the pathophysiological process of AR. To validate the accuracy of microarray analysis, we further randomly chose and validated 4 lncRNAs and 2 mRNAs from the differentially expressed RNAs by qRT–PCR. The consistency of our verification results with those of the microarray analysis strongly suggests the reliability of the microarray results.

The functions of the lncRNAs have not yet been fully annotated and the most common method for their functional prediction is through referring to the functional annotations of their co-expressed mRNAs [13]. As shown in Fig. 3, the differentially expressed lncRNAs are co-expressed with hundreds of mRNAs, which may play a vital role in the pathogenesis and development of AR, such as MUC7, IL 33, THY1, and CXCL12. We predicted the functions of the lncRNAs by GO/KEGG enrichment analysis of these co-expressed mRNAs. The most enriched GO annotations are involved in positive regulation of the integrin biosynthetic process, cell adhesion, focal adhesion, inflammatory response, extracellular matrix, T cell receptor complex, cell junction, and intracellular calcium activated chloride channel activity. Some of these functions are known to be involved in the pathogenesis of AR, such as immune cell activation, inflammatory cell migration, and inflammatory response. KEGG pathway analysis also showed that the co-expressed mRNAs regulated some signaling pathways involved in the activity and function of immune cells, including protein processing in the endoplasmic reticulum, protein export, MAPK signaling pathway, and leukocyte transendothelial migration. Interestingly, recent studies have shed light on these biological processes, molecular functions, cellular components, and signaling pathways associated with AR [11, 26, 28, 29].

Due to the variety of functions of lncRNAs, their molecular regulatory mechanism remains unknown [30]. Previous studies have reported that lncRNAs regulate the transcription of nearby genes in a cis-regulatory manner by recruiting remodeling factors to local chromatin [31]. In this study, we explored the cis-regulatory relationships between the differentially expressed lncRNAs and their co-expressed mRNAs (Table 3). We found that the expression of tight junction proteins and chemokines, such as CLDN1 and CXCL12, were cis-regulated by lnc-TMEM207-2 and lnc-CXCL12-4, respectively. When combined with our KEGG pathway analysis, the differentially expressed CLDN1, CXCL12, and THY1 are involved in leukocyte transendothelial migration. The comprehensive analytical result provides additional information concerning immune cell migration mediated by lncRNAs in the pathogenesis of AR. We also constructed the lncRNA–TF and lncRNA–TF–mRNA network based on the results of trans-regulatory analysis. The core network (Fig. 5) shows that TFs, including STAT2, GATA2, GATA3, and ZBTB7A, regulate lncRNA expression in AR. The expression of SAMD9 is regulated by STAT2, which plays a role in regulating cell proliferation and apoptosis. The proteins encoded by GATA2 and GATA3 play essential roles in regulating the transcription of genes involved in the development and proliferation of hematopoietic cell lineages and T cells. Diseases associated with ZBTB7A include photosensitive epilepsy and lymphoma. Thus, trans-regulatory analysis provides another way to predict the functions of lncRNAs in the pathogenesis of AR.

Besides genetic and lifestyle-related factors, AR is also affected by the composition of inhaled air. The respiratory epithelial cells may mediate parts of the innate and adaptive immunity by their antigen presentation, phagocytosis, cytokine secretion, and pattern recognition abilities [32]. The epithelial surface of the respiratory tract is the “first battlefield” of allergic inflammation, where the epithelial cells interact with the inhaled allergens and trigger inflammatory cascade reactions. A recent study found that CXCL12 and the chemokine receptor CXCR4 were critical components of the inflammatory processes involved in a murine model of allergic airway disease [26]. Upon interaction with CXCR4, CXCL12 can result in the most efficacious chemoattraction of T lymphocytes. In the present study, we examined the epithelial responses to allergen exposure using a cell culture model and demonstrated that OVA/HDM exposure induced the expression of lnc-CXCL12-4, CXCL12, and CXCR4 in BEAS-2B within a short time after exposure compared to untreated cells. This is consistent with our previous clinical observations. In nasal polyps from patients with AR, the expressions of lnc-CXCL12-4, CXCL12, and CXCR4 were increased significantly compared to those from nasal polyps without AR. Taken together, these data support the potential importance of lnc-CXCL12-4 and the CXCL12/CXCR4 axis in the immune responses and inflammation in AR.

## Conclusions

A series of aberrantly expressed lncRNAs may participate in the regulation of target protein-coding genes involved in the biological pathways related to AR in cis- and trans-regulatory manners. On the basis of these findings, we propose that the CXCL12/CXCR4 axis plays a very significant role in the inflammatory process of AR, which is regulated by lnc-CXCL12-4. Leukocyte transepithelial migration may be a potential target for lncRNAs to regulate allergic inflammation.

## Supplementary Information

**Additional file 1: Table S1.** Clinical characteristics of AR and non-AR patients.

**Additional file 2: Table S2.** The differentially expressed mRNAs and lncRNAs.

## Data Availability

The datasets generated and/or analysed during the current study are available in NCBI Gene Expression Omnibus, http://www.ncbi.nlm.nih.gov/geo, GEO Submission: GSE159415.

## Abbreviations

AR: Allergic rhinitis
FC: Fold change
FDR: False discovery rate
GO: Gene ontology
HDM: House dust mite
KEGG: Kyoto Encyclopedia of Genes and Genomes
LncRNAs: Long noncoding RNAs
OVA: Ovalbumin
PCC: Pearson’s Correlation Coefficient

## References

[1] Cheng L, Chen J, Fu Q, He S, Li H, Liu Z (2018). Chinese society of allergy guidelines for diagnosis and treatment of allergic rhinitis. Allergy Asthma Immunol Res.

[2] Wheatley LM, Togias A (2015). Clinical practice. Allergic rhinitis N Engl J Med.

[3] Meltzer EO (2016). Allergic rhinitis: burden of illness, quality of life, comorbidities, and control. Immunol Allergy Clin N Am.

[4] Meng Q, Li P, Li Y, Chen J, Wang L, He L (2019). Broncho-vaxom alleviates persistent allergic rhinitis in patients by improving Th1/Th2 cytokine balance of nasal mucosa. Rhinology.

[5] Zhu X, Wang X, Wang Y, Zhao Y (2020). Exosomal long non-coding RNA GAS5 suppresses Th1 differentiation and promotes Th2 differentiation via downregulating EZH2 and T-bet in allergic rhinitis. Mol Immunol.

[6] Wang Y, Sha J, Wang H, An L, Liu T, Li L (2018). P-FN12, an H4R-based epitope vaccine screened by phage display, regulates the Th1/Th2 balance in rat allergic rhinitis. Mol Ther Methods Clin Dev.

[7] Alexander RP, Fang G, Rozowsky J, Snyder M, Gerstein MB (2010). Annotating non-coding regions of the genome. Nat Rev Genet.

[8] Fatica A, Bozzoni I (2014). Long non-coding RNAs: new players in cell differentiation and development. Nat Rev Genet.

[9] Kung JT, Colognori D, Lee JT (2013). Long noncoding RNAs: past, present, and future. Genetics.

[10] Ma Z, Teng Y, Liu X, Li J, Mo J, Sha M (2017). Identification and functional profiling of differentially expressed long non-coding RNAs in nasal mucosa with allergic rhinitis. Tohoku J Exp Med.

[11] Ma Y, Shi L, Zheng C (2018). Microarray analysis of lncRNA and mRNA expression profiles in mice with allergic rhinitis. Int J Pediatr Otorhinolaryngol.

[12] Qian X, Shi S, Zhang G (2019). Long non-coding RNA antisense non-coding RNA in the INK4 locus expression correlates with increased disease risk, severity, and inflammation of allergic rhinitis. Medicine (Baltimore).

[13] Guttman M, Amit I, Garber M, French C, Lin MF, Feldser D (2009). Chromatin signature reveals over a thousand highly conserved large non-coding RNAs in mammals. Nature.

[14] Guttman M, Donaghey J, Carey BW, Garber M, Grenier JK, Munson G (2011). lincRNAs act in the circuitry controlling pluripotency and differentiation. Nature.

[15] Luo M, Li Z, Wang W, Zeng Y, Liu Z, Qiu J (2013). Long non-coding RNA H19 increases bladder cancer metastasis by associating with EZH2 and inhibiting E-cadherin expression. Cancer Lett.

[16] Spurlock CF, Crooke PS, Aune TM (2016). Biogenesis and transcriptional regulation of long noncoding RNAs in the human immune system. J Immunol.

[17] Villegas VE, Zaphiropoulos PG (2015). Neighboring gene regulation by antisense long non-coding RNAs. Int J Mol Sci.

[18] Hu S, Wang X, Shan G (2016). Insertion of an Alu element in a lncRNA leads to primate-specific modulation of alternative splicing. Nat Struct Mol Biol.

[19] Warburton AJ, Boone DN (2017). Insights from global analyses of long noncoding RNAs in breast cancer. Curr Pathobiol Rep.

[20] Nelson BR, Makarewich CA, Anderson DM, Winders BR, Troupes CD, Wu F (2016). A peptide encoded by a transcript annotated as long noncoding RNA enhances SERCA activity in muscle. Science.

[21] Wang Y, Qian CY, Li XP, Zhang Y, He H, Wang J (2015). Genome-scale long noncoding RNA expression pattern in squamous cell lung cancer. Sci Rep.

[22] Zhang J, Zhu Y, Wang R (2018). Long noncoding RNAs in respiratory diseases. Histol Histopathol.

[23] Shan K, Liu C, Liu BH, Chen X, Dong R, Liu X (2017). Circular noncoding RNA HIPK3 mediates retinal vascular dysfunction in diabetes mellitus. Circulation.

[24] Kim HJ, Nam YR, Nam JH (2018). Flos magnoliae inhibits chloride secretion via ANO1 inhibition in calu-3 cells. Am J Chin Med.

[25] Liu Z, Yang X, Liu X, Mu Y, Wang L, Song X (2021). Analysis of expression of ILC2 cells in nasal mucosa based on animal model of allergic bacterial infection rhinitis. J Infect Public Health.

[26] Gonzalo JA, Lloyd CM, Peled A, Delaney T, Coyle AJ, Gutierrez-Ramos JC (2000). Critical involvement of the chemotactic axis CXCR4/stromal cell-derived factor-1 alpha in the inflammatory component of allergic airway disease. J Immunol.

[27] Gluck J, Rymarczyk B, Rogala B (2012). Serum IL-33 but not ST2 level is elevated in intermittent allergic rhinitis and is a marker of the disease severity. Inflamm Res Off J Eur Histamine Res Soc.

[28] Aune TM, Spurlock CF (2016). Long non-coding RNAs in innate and adaptive immunity. Virus Res.

[29] Hu G, Tang Q, Sharma S, Yu F, Escobar TM, Muljo SA (2013). Expression and regulation of intergenic long noncoding RNAs during T cell development and differentiation. Nat Immunol.

[30] Guttman M, Rinn JL (2012). Modular regulatory principles of large non-coding RNAs. Nature.

[31] Guenzl PM, Barlow DP (2012). Macro lncRNAs: a new layer of cis-regulatory information in the mammalian genome. RNA Biol.

[32] Toppila-Salmi S, van Drunen CM, Fokkens WJ, Golebski K, Mattila P, Joenvaara S (2015). Molecular mechanisms of nasal epithelium in rhinitis and rhinosinusitis. Curr Allergy Asthma Rep.

[33] Kanehisa M, Furumichi M, Sato Y, Ishiguro-Watanabe M, Tanabe M (2021). KEGG: integrating viruses and cellular organisms. Nucleic Acids Res.

