# Supplementary material for: Genome-wide analysis of long noncoding RNA expression profile in nasal mucosa with allergic rhinitis
Source: BMC Med Genomics. 2021 Apr 9;14:100. doi: 10.1186/s12920-021-00949-4 (PMC8033732; doi:10.1186/s12920-021-00949-4)
Supplement: Supplementary file 1 — Additional file 1: Table S1. Clinical characteristics of AR and non-AR patients. [file 12920_2021_949_MOESM1_ESM.docx]

**Title**: Genome-wide analysis of long noncoding RNA expression profile in nasal mucosa with allergic rhinitis

Xian Wei^1,†^,Man Xu^1,†^,Chao Wang^1^, Shengjian Fang^1^, Yu Zhang^1^, Weihua Wang^1,2^*

1. Department of Otolaryngology-Head and Neck Surgery, Shanghai East Hospital, Tongji University School of Medicine, Shanghai, P.R. China

2. Department of Otolaryngology-Head and Neck Surgery, Shanghai East Hospital Ji’an Hospital, Ji’an, Jiangxi, P.R. China

**Additional file 1: Table S1** **Clinical characteristics of AR and non-AR patients**

| AR Non-AR | | | | | | | | |
| --- | --- | --- | --- | --- | --- | --- | --- | --- |
|  | Age | Sex | Allergen species |  |  | Age | Sex | Allergen species |
|  | 33 | Male | HDM |  |  | 30 | Male | - |
|  | 40 | Male | HDM /Cat hair |  |  | 35 | Male | - |
|  | 53 | Female | HDM |  |  | 32 | Male | - |
|  | 68 | Female | HDM /Pollen |  |  | 68 | Male | - |
|  | 29 | Female | HDM |  |  | 59 | Female | - |
|  | 30 | Male | HDM |  |  | 26 | Female | - |
|  | 28  29 | Female  Male | HDM  HDM |  |  | 46  57  43  29 | Male  Male  Female  Male | -  -  -  - |

HDM: house dust mite.
